# Food Consumption Patterns in Romania during the COVID-19 Pandemic

**DOI:** 10.3390/foods10112712

**Published:** 2021-11-05

**Authors:** Diana E. Dumitras, Rezhen Harun, Felix H. Arion, Daniel I. Chiciudean, Eniko Kovacs, Camelia F. Oroian, Andra Porutiu, Iulia C. Muresan

**Affiliations:** 1 Department of Economic Sciences, University of Agricultural Sciences and Veterinary Medicine Cluj-Napoca, 3-5 Manastur Street, 400372 Cluj-Napoca, Romania; ddumitras@usamvcluj.ro (D.E.D.); felixarion@usamvcluj.ro (F.H.A.); daniel.chiciudean@usamvcluj.ro (D.I.C.); eniko.kovacs@usamvcluj.ro (E.K.); camelia.oroian@usamvcluj.ro (C.F.O.); andra.porutiu@usamvcluj.ro (A.P.); 2 Department of Agribusiness and Rural Development, College of Agricultural Sciences Engineering, University of Sulaimani, Sulaimani 5100, Iraq; rezhen.rashid@univsul.edu.iq; 3 National Institute for Research and Development of Optoelectronics Bucharest INOE 2000, Research Institute for Analytical Instrumentation Subsidiary, 67 Donath Street, 400293 Cluj-Napoca, Romania

**Keywords:** consumer behavior, COVID-19 pandemic, food changing habits, shopping behavior

## Abstract

Food consumption behavior during the COVID-19 pandemic has changed worldwide as a consequence of the restrictions imposed by law and/or due to the fear of contamination. Although some similarities are found among countries, there are still many particularities for each nation. The present study focused on Romanian consumers and their consumption behavior related to four main food categories: fruits and vegetables, meat and meat products, bread and bakery products, and milk and milk products. Frequency of buying, shopping habits, place of purchase, and concerns related to the place of purchasing food products during the COVID-19 pandemic were analyzed in comparison with the pre-COVID-19 period using descriptive statistics and inferential statistics regarding an online survey. Three types of behavior were identified as being related to the frequency of buying and to organizing a shopping list (less often, no change, more often). Two groups of consumers were identified as being related to the place of purchasing food: people with the same habits and people with new habits. Concerns related to the location of the stores and to the choice of buying directly from producers were also investigated using the ordered logistic regression. The empirical study revealed the new consumption patterns with a reflection on future trends.

## 1. Introduction

The unexpected and rapid spread of the COVID-19 pandemic pushed people to experience new life habits. To restrain the virus from spreading, people had to comply with several restrictions imposed by law, with a large part of the population being forced to stay at home. In Romania, as a response to the evolution of the epidemic, the government declared a state of emergency on the 16th of March 2020 [1] for a duration of 30 days, which was later expanded. Temporary legislation was enacted by military ordinances. A national lockdown was instituted on 29 March 2020, through the military ordinance no. 3 [2], which involved several constraint measures, such as restricted range of movement; remote work (where applicable); temporary closure of restaurants, cafes, shopping centers (except for the sale of food), and farmers’ markets; reduced opening hours of grocery stores, and other restrictions. On the 15th of May 2020, a state of alert was established [3], through which some of the restrictions were still kept (e.g., reduced opening hours of grocery stores, closed farmers’ markets).

All these led to changes in the everyday buying behavior of the majority of consumers, as well as in the lifestyle and eating habits in Romania [4,5,6] and in other countries from the South-Eastern European region [7,8,9]. To be able to take correct actions and adopt appropriate policy measures, we must understand how people changed their food consumption behavior during the COVID-19 pandemic as compared to the pre-pandemic period, as pointed by Jansen et al. [10]. Our present knowledge of the transition to new patterns of food consumption, however, is limited in the South-Eastern European region.

In this context, the following research questions are raised regarding consumers’ behavior after the state of emergency as compared to the pre-COVID-19 period in Romania: How has the frequency of buying food products changed since COVID-19 pandemic? How was affected the organization of grocery shopping since COVID-19 pandemic? To what extent did the location of stores influence consumers’ decision to choose where to purchase food products? An empirical study investigating the changes in food consumption behavior that occurred during the COVID-19 pandemic among consumers from the North-West Development Region of Romania was used to answer the research questions addressed. The specific objectives referred to examining consumers’ behavior after the state of emergency as compared to the pre-COVID-19 period in terms of the following: frequency of buying food, shopping habits, and place of purchase. The new consumption patterns were analyzed in the case of four main food categories (fruits and vegetables, meat and meat products, bread and bakery products, milk and milk products) with a reflection on future trends. The study’s findings can provide valuable country-specific elements for developing knowledge-based policy measures in Romania, filling the research gap that exists on the topic.

The paper is structured in six sections. The introduction is followed by a literature review on consumers’ behavior related to food products during COVID-19 pandemic. Section 3 presents the methodology used, while results are presented in Section 4. Discussions are provided in Section 5. The work ends with conclusions and implications of the study, presented in Section 6.

## 2. Literature Review

A large number of studies have been conducted worldwide to better understand the pandemic’s impact on consumer behavior, as it varies from country to country, depending on the progression of the infections and on policies. For instance, in China, the first country that reported the presence of COVID-19, among the first observations regarding the shift in youths’ dietary patterns were decreases in the consumption of fresh vegetables and fruits, meat, rice, and dairy, and increases in wheat and other basic feed, as well as preserved vegetables [11]. A study conducted by Wang et al. [12] showed that the dairy industry, both in China and in the USA, was significantly impacted during the lockdown in terms of transportation, workforce, operating costs, and milk prices at the farm. Moreover, both countries were forced to discard milk due to the lack of buyers. The same phenomenon occurred in the United Kingdom and Canada [12,13]. In contrast, in other countries, such as Colombia, Chile, and Brazil, an increase in milk demand was observed [14]. In Italy, one of the most affected countries worldwide [15], the eating behavior of the population was severely modified by the imposed restrictions. Fanelli’s [16] findings classified the impact of the pandemic into three categories: changes in food purchasing, dietary patterns, and food-related habits. In the same context, Di Renzo et al. [17] studied the eating habits of the population aged between 12 and 86 years under the immediate effects of the lockdown, finding that there was an increase in the consumption of home-prepared food, vegetables, and white meat, with 15% of the interviewees purchasing fruits and vegetables from farmers or organic suppliers, and a decrease of fresh fish, pastries, and delivery food. The majority of the Italian respondents purchased food from supermarkets, followed by grocery stores, local markets, and then online. Young Italians’ shopping habits were adjusted to the new conditions by making shopping lists and carefully planning their meals [18]. In the United Kingdom, Snugs and McGregor [19] conducted a study interviewing 240 adults and analyzed their food preferences with an emphasis on food preparation and food decision making. The respondents’ feedback can be grouped in two clusters: deliberate choice of healthy lifestyles, or options limited by unhealthy habits or financial constraints. Filimonau et al. [20] studied the impact of the pandemic on food consumption both at home and away, and their qualitative results showed that the population developed a high interest in healthy home-cooked food coming from local producers, but there was no increase in organic food consumption in households. Concerning the frequency of buying, the majority of the participants in the survey reported that their grocery shopping frequency decreased. In Poland, Sidor and Rzymski [21] investigated the eating habits during the quarantine and found that a large number of people changed their food consumption patterns, eating more snacks, meat, and dairy and less vegetables and fruit than before the pandemic. Similar results were found by Deschasaux-Tanguy et al. [22], reporting a higher intake of snacks and a lower consumption of fruits and vegetables. A study conducted by Hassen et al. [23] examined the pandemic’s early effects on food purchase and consumption habits in Russia, revealing that consumers reduced the frequency of shopping trips and the quantity of purchased products in order to minimize their exposure to risk due to the fear of the virus, which was also evidenced by Reznik et al. [24]. Moreover, people adopted healthier consumptions patterns, consuming more fruits and vegetables and less snacks and pastries [17,24]. Food purchase behavior was also analyzed by Ellison et al. [25], revealing that purchasing occurred mostly in grocery stores, followed by corner stores, with online shopping showing an increasing trend.

Furthermore, a limited number of studies have been carried out in the South-Eastern European region [7,8,9] regarding the changes in consumption habits associated with the pandemic. In Romania, focusing on a precise territory, namely, Suceava, Butu et al. [4] studied consumers’ behavior during the COVID-19 pandemic regarding fresh vegetables from local growers, finding that although the COVID-19 crisis significantly changed the buying behavior of fresh products directly from local producers, it left the selection method of fresh vegetables unchanged. Burlea-Schiopoiu et al. [5] found that responsible food shopping and food-related waste behavior in young people was positively influenced by the COVID-19 crisis. In line with internal values, ethical norms, budget, and needs, food shopping under COVID-19 became a less compulsive process due to behavioral control factors such as shopping routines, reuse of leftovers, meal planning, and cooking. After the short-term price increase due to product shortage and to logistical restrictions, solutions such as online shopping and product adaptation to a longer storage time re-established the balance. The consumption of organic fruits and vegetables and other healthy products increased in response to a higher caloric intake during the lock-down periods and to a rising concern for maintaining immunity against the spreading disease.

Interesting findings were also found related to home cooking behavior. Specifically, researchers noticed a change towards home cooking throughout the pandemic [17,20,22,23,26,27,28,29]. Another phenomenon present across many countries during the pandemic was stockpiling of non-perishable food and panic purchases as a preventative measure to the virus exposure and as a reaction to concerns about food shortages [23,30,31,32,33,34,35,36].

The findings of the above-mentioned research studies show that the COVID-19 pandemic had a significant impact on consumers’ eating habits with regard to dietary preferences and food purchasing decision.

## 3. Materials and Methods

A questionnaire was used to collect data regarding consumption habits during the pre-COVID-19 period and after the COVID-19 state of emergency. To capture the changes in consumers’ behavior, the questionnaire included the same set of questions for the two moments. The questionnaire was developed on the basis of other studies [37,38]. The collected data for this research were grouped into 3 parts: (i) consumption habits (frequency of buying food products and place of purchase), (ii) shopping habits (4 items evaluated on a Likert-type scale from 1 to 5, where 1 means very rare and 5 means very often), and (iii) socio-demographic characteristics (gender, age category, level of education, monthly net household income, work category, and presence of children in the household). Four food categories were considered: fruits and vegetable, meat and meat products, bread and bakery products, and milk and milk products. This approach allowed a point-by-point comparison of the responses on the basis of which were identified the groups of consumers. The comparison between the two moments (pre-COVID-19 period and after the COVID-19 state of emergency) allowed for the identification of consumers who have not changed their behavior and of consumers who changed their behavior. To ensure face and content validity of the instrument, we asked three experts in the field to verify the questionnaire [39,40]. The pilot test also helped to check the reliability of the items measured on a 5-point scale. Chronbach’s alpha was 0.74, which means that the scale in this study is reliable [41].

Data were collected online from May to October 2020, with the questionnaires being distributed using social media. Respondents were informed about the aim of the study and the protection of the GDPR data at the beginning of questionnaire. In order to achieve 95% confidence interval and ±3.5% sampling error, we determined a sample size of 784 respondents. Respondents were selected on the basis of their residence county and age by using the convenience sampling until the required sample size was reached [42]. In total, 859 responses were validated from a total of 1103 collected questionnaires from the six counties of the North-West Development Region of Romania: Bistrita-Nasaud, Bihor, Cluj, Maramures, Satu-Mare, and Salaj.

Data were analyzed using descriptive statistics, inferential statistics (chi-squared test, Fisher’s exact test), and regression analysis (the ordered logit regression) [43,44]. Data normality was checked using the Shapiro–Wilk test. *p*-value of <0.05 was considered statistically significant. The post hoc Bonferroni was used to compare differences in case of significance. STATA version 15.0 (StataCorp, College Station, TX, USA) was used for all analyses.

## 4. Results

### 4.1. Respondents Profile

The socio-demographic characteristics of the respondents are presented in Table 1. The sample is composed of 61.1% female and 38.9% male respondents. Respondents’ distribution in terms of age was in accordance with the original population, with about half being between 18 and 39 years old. In terms of the level of education, the majority had a university degree, with a slightly lower percentage in the case of Bihor County. Monthly net household income varied. A total of 90.2% of respondents belonged to households of up to four members, from which 8% lived alone. There were 51.3% families with children, from which 7.7% had more than two children. The sample was representative of all working categories, with the main category being those who are employed. Statistically significant differences were found among counties in terms of the socio-demographic characteristics (*p* < 0.01).

### 4.2. Changes in Consumption Habits during the COVID-19 Pandemic

#### 4.2.1. Frequency of Buying Food Products

**Figure 1:**
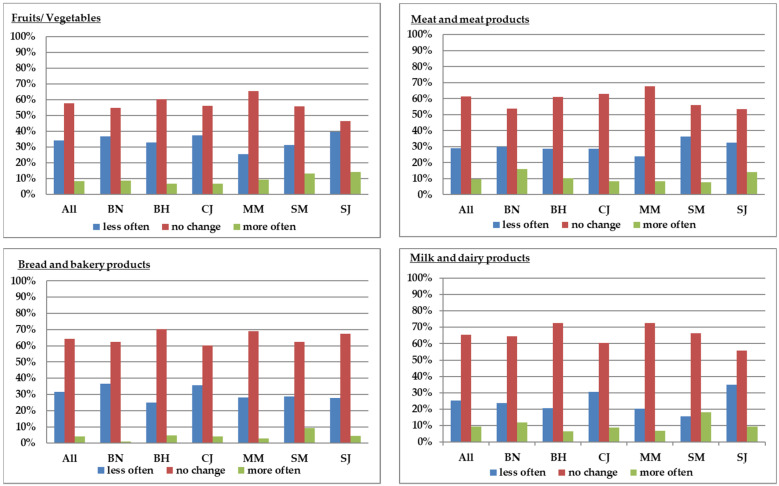
Frequency of buying food during the pandemic as compared to the pre-COVID-19 period (BN: Bistrita-Nasaud, BH: Bihor, CJ: Cluj, MM: Maramures, SM: Satu-Mare, SJ: Salaj).

To address the first research question regarding the way in which the frequency of buying food products changed since the COVID-19 pandemic, we split respondents into three groups on the basis of the frequency of buying food after the state of emergency as compared to the pre-COVID-19 period. One group was composed of consumers who decided to buy less often after the state of emergency, another group comprised people who maintained their shopping habits in terms of frequency, and the third group consisted of consumers who bought more often than before. The identification of different buying behaviors allowed for a better understanding of the changes that occurred in each county and for each product category, as pictured in Figure 1. The most affected food category was fruits and vegetables, with about 58% of respondents not changing their buying behavior in terms of frequency, followed by meat and meat products with 61%, bread and bakery products with 64%, and milk and dairy products with 66%. The percentage of people who bought less often varied depending of the food category and county: 25.4% to 39.5% for fruits and vegetables, 23.9% to 36.4% for meat and meat products, 28.2% to 36.6% for bread and bakery products, and 15.6% to 34.9% for milk and dairy products. The percentage of people who bought more often was the smallest, with up to 14% in the case of fruits and vegetables, 16.1% in the case of meat and meat products, 11.8% for milk and dairy products, and 9.1% for bread and bakery products. When comparing the habits among counties, we found a statistical difference for milk and dairy products (*p* < 0.01), with a higher percentage of consumers who bought more often being encountered in Bistrita-Nasaud and Satu-Mare counties. Changes were similar for the other food categories (*p* > 0.05).

#### 4.2.2. Shopping Habits

**Figure 2:**
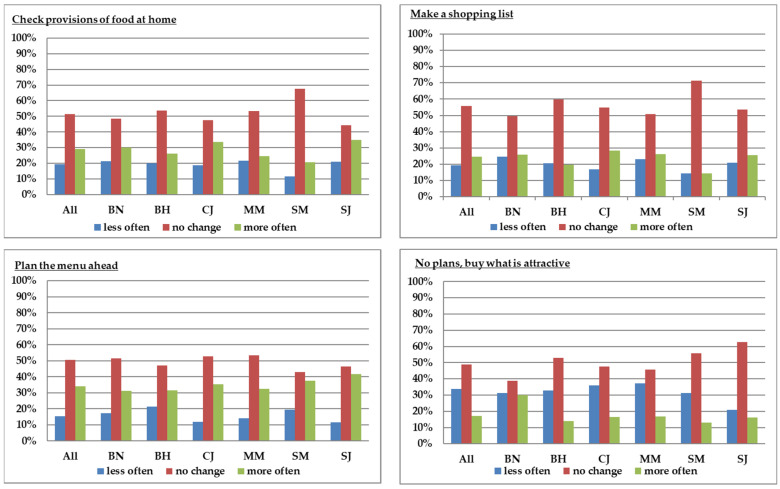
Shopping habits during the pandemic as compared to the pre-COVID-19 period (BN: Bistrita-Nasaud, BH: Bihor, CJ: Cluj, MM: Maramures, SM: Satu-Mare, SJ: Salaj).

The second research question referred to the organization of grocery shopping since the COVID-19 pandemic. Consumers’ habits related to checking provisions of food at home before shopping, making a shopping list ahead or not, and planning the menu for the next period were analyzed to understand the way of organizing the shopping after the state of emergency as compared to the pre-COVID-19 period (Figure 2). Three groups of consumers were identified in this respect: people who acted less often after the state of emergency, people who acted the same way, and people who acted more often. About 20% of respondents declared that after the state of emergency, they checked less often the provisions of food at home before going for food shopping, while about 30% checked the provisions more often. Habits related to writing a shopping list changed for 46% of respondents, with the rest choosing either to make a list more often (14.3% to 28.5%) or less often (14.3% to 24.7%). About 35% of consumers stated that they planned the menu ahead more often than before, while only 15% planned less often. The choice to buy more often what is attractive without previous plans seemed to be the case for a relatively small percentage of consumers (17%). Statistical difference among counties were found only for the statement “I make no plans and buy what is attractive” (*p* < 0.01). A higher percentage of consumers who buy more often what is attractive was registered in Bistrita-Nasaud county as compared to the other counties.

#### 4.2.3. Place of Purchasing Food Products

**Figure 3:**
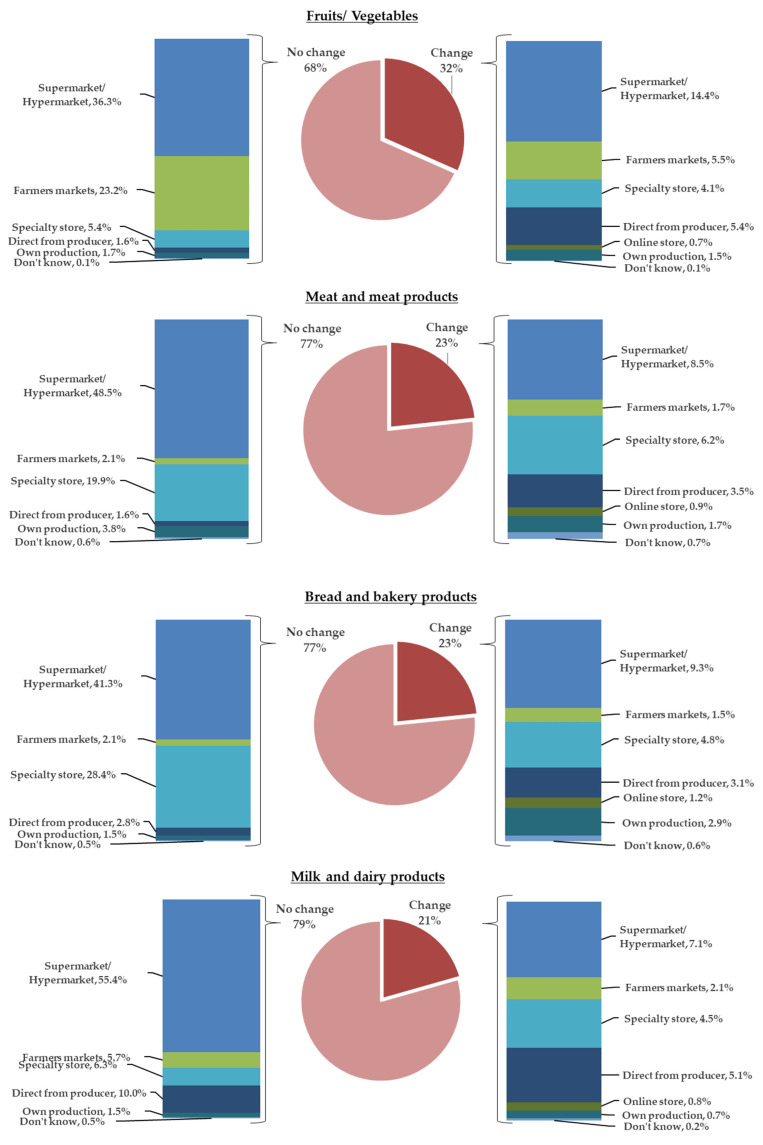
Place of purchase habits during the pandemic.

The third research question explored to what extend the location of stores had influenced consumers’ decision to choose from where to purchase food products. When analyzing the behavior related to the choice of the place of purchasing food products after the COVID-19 state of emergency, we identified two groups of consumer buying behavior. The first group is composed of people who decided to buy from the same type of selling points, and the second group consists of people who switched to different types of selling points. Figure 3 shows for each analyzed food category the places of purchase chosen by consumers for the above-mentioned groups. About 32% of consumers chose to buy fruits and vegetables from places other than where they were used to buying them from, 23% in the case of meat and bread, and 21% in the case of milk and dairy products. Supermarkets prevailed for all four categories, followed by specialty stores and direct producers. Online stores were not popular in the pre-COVID-19 period and became an option for a very small group of consumers after the state of emergency (about 1%). In general, people switched from farmers’ markets and specialty stores to supermarkets. Less than 2% consumed fruits and vegetables, meat, and dairy products from their own production, and slightly more chose to cook their own bread at home (about 3%). When comparing the habits of the two groups among counties, we found that changes were similar for the all food categories (*p* > 0.05).

The two groups of consumers were further analyzed in terms of their concern related to the place of purchase, that is, the choice of buying food products at shops near the house and the choice of buying directly from producers rather than from bigger stores such as supermarkets or hypermarkets. The analysis consisted in understanding to what extent the level of concern (scale from 1 to 5, where 1 = to a very little extent and 5 = to a very great extent) is closely linked to respondents’ characteristics (gender, age, education, income, children in the household) with the aid of the ordered logistic model. The concern related to buying food products at shops located near the house was not influenced by income, education level, nor by the presence of children (*p* > 0.05) (Table 2). The group of consumers who chose not to change the place of purchase during the pandemic was influenced only by their gender (*p* < 0.05). Male respondents were less concerned about the purchase place in the case of all four food products. When analyzing the group of consumers who chose to change the place of purchase after the COVID-19 state of emergency, we identified influencing factors only for two food categories: fruits and vegetables, and meat and meat products. Respondents of ages between 40 and 59 years were more concerned about buying fruits, vegetables, and meat near their house, perhaps being more conscious about assuring a diversified and healthy diet. Moreover, consumers older than 60 were more concerned about where to buy fruits and vegetables after the state of emergency, with this group also being concerned about a healthy diet. The insignificant models in the case of bread and dairy products can be explained by the presence of specialty shops in the neighborhoods, thus not representing a concern.

The concern related to buying food products directly from producers is not influenced by gender, income, nor by education level (*p* > 0.05) (Table 3). The group of consumers who chose not to change the place of purchase after the COVID-19 state of emergency was influenced by their age (*p* < 0.05). In the case of all food products, consumers older than 40 years were more concerned, being conscious about the importance of eating fresh products, most likely being willing to keep the source based on previous experience. In addition, families with children were more concerned about buying fruits, vegetables, and meat directly from producers (*p* < 0.05). The analysis of the group that chose to change the purchase place indicates that age is not an influencing factor in the case of milk and dairy products (*p* > 0.05), while it is in the case of the other three food categories (*p* < 0.05). However, consumers with children are more concerned about buying milk directly from producers (*p* < 0.05).

## 5. Discussion

The present study reveals important findings related to consumers’ behavior in terms of the frequency of buying food, shopping habits, and place of purchase after the state of emergency as compared to the pre-COVID-19 period. First, the analysis of the frequency of buying food products during the COVID-19 pandemic led to three distinct consumer buying behaviors, each with its particularities with regard to the four food categories: fruits and vegetables, meat and meat products, bread and bakery products, and milk and milk products. The three groups are as follows: people who bought less often, people with the same habits, and people who bought more often. Changes occurred for all four food categories, with the highest percentage of people with different behavior in the case of fruits and vegetables and the least percentage in the case of bread and bakery products. The tendency was to buy less often in the case of consumers who changed their habits, even though there was a small group that declared they buy more often. Other studies have also found a lower frequency of grocery shopping [45,46]. The behavior of the group who bought more often food products can be explained by the general change of lifestyle during the pandemic with people working from home. Although at the national level, a small percentage of people worked from home (2.5% in 2020 according to Eurostat database [47]), changes were seen due to the overall pandemic situation. This was noticed for all products except for bread and bakery products, a finding that can be explained due to the general habits of Romanian consumers to buy bread more times per week and some even daily. Bread represents one of the main food products consumed [48], with Romania being in the top countries with the highest wheat consumption per capita [49].

Second, the analysis focused on understanding whether the organization of grocery shopping has changed due to the COVID-19 pandemic. The findings revealed three types of behavior: people who acted less often, people who acted the same way, and people who acted more often. About half of respondents behaved differently after the state of emergency as compared to the pre-COVID-19 period. Changes occurred in terms of checking the food provisions, making a list, and planning the menu prior the shopping session. In some cases, these habits occurred more often, in other cases more seldom. We expected to find changes in this respect due to the restrictions imposed by the state, such as limited opening hours and filling out a form to leave the house [1], but also due to the fear of contamination and spread of the virus that was present among Romanians regardless of age [6]. In fact, this fear has been experienced worldwide [24,50,51,52] and has affected the access to food [53,54]. The fear of limited access to food encourages peoples to buy larger quantities than usual [55], thus assuring food provisions in their household. Changes in behavior related to planning a menu also occurred due to the changes in eating habits. People who were used to eating lunch and/or dinner outside the house were forced to change their habits during the lockdown towards home cooking [29] and/or online delivery [56]. Cooking at home was seen as one of the positive effects of pandemic [57], with people developing or regaining cooking skills as they had more time to prepare food [29,58]. Moreover, home cooking is considered a better way to assure a healthy diet than eating outside the home [58,59].

The third research direction explored the choice of the place of purchasing food products during the COVID-19 pandemic as a key point in understanding consumers’ behavior due to the restrictions imposed [1], which forced people to reconsider their habits and due to the fear of contamination and spread of the virus [6]. Two groups of consumer buying behavior related to the place of purchasing food were identified: people with same habits and people with new habits. Changes occurred by switching from one type of selling point to another and varied among food categories. Many consumers chose to buy from supermarkets, their choice being probably based on the convenience of the “one-stop shop” and strict hygiene measures taken. Specialty stores are also popular, especially for meat, dairy, and bakery products, such stores being located in the neighborhood of the consumers. Own production was reported in a small percentage, most probably only in the case of people who had the possibility to start or spend more time gardening. It is possible that their choice had benefits not only on their health, but also on their well-being, as it was proven in a study conducted in Italy that the contact of people with nature during the COVID-19 lockdown had positive impacts on distress [60]. Online stores started to become an option also for food products, even though it was for a small percentage. Until the COVID-19 pandemic, online shopping was more popular for non-food products and highly depended on the ease of using the web platform and on the reviews [61]. Although e-commerce is not as popular in Romania as in other EU countries [62], it registered an increase due to the pandemic, as reported in the Eurostat database [63]. Buying directly from producers also became fairly popular as compared to the previous period, for reasons such as providing fresh and healthy food products or supporting local producers. In this sense, social media played an incredible role in supporting the small local producers who gathered their forces and created social groups to promote their products and to offer home delivery on a regular basis or on demand. This practice is currently continuing, even though people have the possibility of switching back to their old habits as shopping restrictions have been lifted and farmers’ markets have opened. It appears that people have learned to appreciate the local producers of their fresh and healthy food products more.

With respect to the concerns related to the location of the stores and to the choice of buying directly from producers, results indicate that male respondents belonging to the group who did not decide to change the purchase places were found to be less concerned about the how far away the stores were located, being more likely to go anywhere to shop, regardless the type of product. On the other hand, consumers older than 40 years showed more responsible behavior in terms of eating habits as they were more concerned about being able to buy directly from producers rather than from larger stores. The same held for families with children when it came to fruits, vegetables, and meat. This behavior is most probably due to the willingness to keep the sources based on previous experiences with the producers, being content with their choices. The tendency towards healthier diets was confirmed also by Brihan et al. [64] who conducted a study on Romanian consumers. Recently, consumers have paid more attention towards learning about the products and their nutritional facts [65,66], perhaps due to the information campaign through mass-media related to the benefits of healthy diets. Thus, it is not surprising that a significant amount of consumers expressed their concern related to the possibility to continue to buy from the same producers. It is worth also mentioning that during the COVID-19 pandemic, the importance of eating fruits and vegetables was highly promoted by the World Health Organization [67], encouraging people towards a healthy diet. The important role of information campaigns related to healthy diets, targeted towards consumers during the COVID-19 pandemic, is acknowledged also by other research [68,69]. The group who decided to change the purchase place was influenced by their age and the presence of children in their family. Specifically, consumers older than 40 years were more concerned with finding new places that offer at least the same experience as the previous location. Families with children also expressed a higher concern when it came to milk purchased directly from producers. Indoor and outdoor farmers’ markets were popular in the entire country, with consumers being used to buy fresh products directly from producers. The fact that farmers’ markets had limited hours or were closed during the lockdown affected people’s habits in great measures.

## 6. Conclusions

Consumers worldwide experienced changes during the COVID-19 pandemic related to food products, with the switch to a new lifestyle being a consequence of the restrictions imposed by law and/or due to the fear of contamination. This study contributes to the reduction of the research gap regarding food consumption patterns during the COVID-19 pandemic in the South-Eastern European region. Specifically, the current work exposed how consumers from the North-West Development Region of Romania changed their behavior during the COVID-19 pandemic as compared to the pre-COVID-19 period.

It would be of high interest to continue the investigation on how long the new consumer behaviors will last and whether any of these changes will prevail when lockdown is ruled out. The fact that the focus was on one development region from Romania may be considered a limitation of the study. The extension of the research area and a comparison analysis among the development regions may reveal some changes. Another limitation may be related to the sampling. The current context limited the access of researchers to persons that have no access to internet and social media, and thus the representativeness of the sample to the original population was affected.

Overall, the study’s findings provide empirical evidence on the new consumption patterns caused by the epidemic and evidence of changing consumption behaviors caused by the COVID-19 pandemic opening reflection on future trends. The results are useful for food processors and retailers, offering them important information that might be used for gaining competitive advantages on the food markets. It is not unneglectable that consumers started to cook more and to pay more attention to the place where they are doing their shopping, preferring more local producers, especially for vegetables and fruits. At the same time, families with children in the household are more concerned in buying products from local producers. This new trend is sustained even today by the new ways of communication between local producers and consumers via social media and their availability to deliver food products either at home or in open air organized places.

## Figures and Tables

**Table 1:** Socio-demographic characteristics of the sample (%).

Characteristic		All (*n* = 859)	County					
			Bistrita-Nasaud (*n* = 93)	Bihor (*n* = 164)	Cluj (*n* = 340)	Maramures (*n* = 142)	Satu-Mare (*n* = 77)	Salaj (*n* = 43)
Gender	Female	61.1	58.1	52.4	67.9	50.7	67.5	69.8
	Male	38.9	41.9	47.6	32.1	49.3	32.5	30.2
			Χ^2^(5) = 21.39, *p* < 0.01					
Age categories	18–29 years	29.0	32.3	21.3	36.5	20.4	24.7	27.9
	30–39 years	24.1	17.2	21.3	27.9	20.4	27.2	25.5
	40–49 years	18.6	20.4	23.8	13.8	23.3	18.2	18.6
	50–59 years	14.9	17.2	18.9	10.6	19.0	15.6	14.0
	60–69 years	13.4	12.9	14.7	11.2	16.9	14.3	14.0
			Χ^2^(20) = 39.07, *p* < 0.01					
Level of education	Up to 8 classes	2.8	11.8	3.7	0.6	1.4	3.9	0.0
	High school diploma	23.2	30.1	21.3	20.0	31.0	19.5	20.9
	University degree	74.0	58.1	75.0	79.4	67.6	76.6	79.1
			Fisher’s Exact *p* < 0.001					
Monthly net household income (RON)	≤2800	19.9	20.4	21.9	15.8	31.7	11.7	18.6
	2801–4200	24.1	31.2	17.1	25.3	16.2	32.5	34.9
	4201–5600	19.7	17.2	15.9	21.5	21.8	19.5	20.9
	≥5601	36.3	31.2	45.1	37.4	30.3	36.3	25.6
			Χ^2^(15) = 38.71, *p* < 0.001					
Work	Student	11.1	21.5	8.5	12.6	4.2	11.7	7.0
	Unemployed	1.7	3.2	3.7	0.6	2.1	0.00	2.3
	Retired	10.0	5.4	6.1	12.1	11.9	15.6	2.3
	Employed	62.7	51.6	66.5	59.7	69.1	62.3	76.8
	Entrepreneur	9.1	8.6	10.3	8.8	9.9	6.5	9.3
	Maternity leave	4.0	8.6	3.7	3.8	2.8	2.6	2.3
	Other (priest, farmer, freelancer)	1.4	1.1	1.2	2.4	0.0	1.3	0.0
			Fisher’s Exact *p* < 0.01					
Children (<18 years) in household	Yes	51.3	69.9	49.4	43.5	55.6	54.5	60.5
	No	48.7	30.1	50.6	56.5	44.4	45.5	39.5
			Χ^2^(5) = 24.16, *p* < 0.001					

Note: RON is Romanian leu; in spring 2020, the average exchange rate was USD 1 = RON 4.42.

**Table 2:** Ordered logistic regression results—dependent variable: “I shop at shops near the house”.

	Fruits and Vegetables		Meat and Meat Products		Bread and Bakery Products		Milk and Dairy Products	
	No change (*n* = 587)	Change (*n* = 272)	No change (*n* = 659)	Change (*n* = 200)	No change (*n* = 658)	Change (*n* = 201)	No change (*n* = 682)	Change (*n* = 177)
	OR (95% CI)	OR (95% CI)	OR (95% CI)	OR (95% CI)	OR (95% CI)	OR (95% CI)	OR (95% CI)	OR (95% CI)
Gender: male	0.55 ** (0.41–0.75)	-	0.72 * (0.545–0.954)	-	0.73 * (0.55–0.97)	-	0.60 ** (0.46–0.80)	-
Age category								
30–39 years	-	1.08 (0.59–1.98)	-	1.36 (0.68–2.70)	-	-	-	-
40–49 years	-	2.23 ** (1.21–4.20)	-	2.00 * (0.96–4.18)	-	-	-	-
50–59 years	-	1.81 * (0.90–3.64)	-	2.55 * (1.18–5.49)	-	-	-	-
≥60 years	-	2.68 ** (1.30–5.51)	-	1.04 (0.37–2.84)	-	-	-	-
Log likelihood	−861.327	−383.47	−967.865	−287.87	−960.325		−1001.200	
Prob > chi^2^	<0.001	<0.01	<0.05	<0.01	<0.05	>0.05	<0.001	>0.05
Pseudo R^2^	0.008	0.016	0.002	0.013	0.002		0.006	

* Significant at the 5% level; ** significant at the 1% level; base category: gender: female; age category: 18–29 years old; OR: odds ratio; CI: confidence interval.

**Table 3:** Ordered logistic regression results—dependent variable: “I choose to shop directly from producers at the expense of supermarkets”.

	Fruits and Vegetables		Meat and Meat Products		Bread and Bakery Products		Milk and Dairy Products	
	No change (*n* = 587)	Change (*n* = 272)	No change (*n* = 659)	Change (*n* = 200)	No change (*n* = 658)	Change (*n* = 201)	No change (*n* = 682)	Change (*n* = 177)
	OR (95% CI)	OR (95% CI)	OR (95% CI)	OR (95% CI)	OR (95% CI)	OR (95% CI)	OR (95% CI)	OR (95% CI)
Age category								
30–39 years	1.08 (0.72–1.62)	1.81 * (0.98–3.32)	1.07 (0.73–1.58)	1.88 (0.94–3.78)	1.42 (0.97–2.07)	0.97 (0.48–1.95)	1.35 (0.93–1.96)	-
40–49 years	1.77 * (1.12–2.78)	1.88 * (1.00–3.51)	1.66 * (1.09–2.54)	2.20 * (1.05–4.64)	1.71 ** (1.14–2.54)	3.55 ** (1.57–8.01)	2.02 ** (1.36–2.99)	-
50–59 years	1.75 * (1.10–2.80)	2.15 * (1.06–4.36)	1.67 * (1.06–2.63)	2.01 * (0.94–4.26)	1.90 ** (1.22–2.98)	1.44 (0.66–3.15)	2.03 ** (1.31–3.17)	-
≥60 years	1.72 * (1.07–2.80)	3.63 ** (1.74–7.54)	1.94 ** (1/25–3.02)	4.12 ** (1.56–10.88)	1.71 * (1.08–2.72)	3.48 ** (1.55–7.79)	2.51 ** (1.60–3.93)	-
Children in household: yes	1.36 * 0.99–1.85)	-	1.36 * (1.01–1.82)	-	-	-	-	2.02 ** (1.18–3.47)
Log likelihood	−883.631	−380.70	−988.754	−286.017	−985.057	−298.901	−1013.427	−253.696
Prob > chi^2^	<0.01	<0.001	<0.001	<0.05	<0.01	0.001	<0.001	<0.01
Pseudo R^2^	0.010	0.030	0.009	0.017	0.006	0.031	0.011	0.013

* Significant at the 5% level; ** significant at the 1% level; base category: age category: 18–29 years old; children in household: no.

## Data Availability

The data presented in this study are available upon request from the corresponding author.
